# Development and validation of single-item experience sampling measures of wellbeing in teens

**DOI:** 10.1038/s41598-026-51642-4

**Published:** 2026-05-21

**Authors:** T. R. A. Kral, C. D. Wilson-Mendenhall, R. Jacobucci, R. J. Davidson, R. Tatar

**Affiliations:** 1 Humin, Madison, WI USA; 2 Center for Healthy Minds, University of Wisconsin –Madison, Madison, WI USA; 3 Department of Psychology, University of Wisconsin –Madison, Madison, WI USA; 4 Department of Psychiatry, University of Wisconsin –Madison School of Medicine and Public Health, Madison, WI USA

**Keywords:** Wellbeing, Adolescents, Teen, Ecological momentary assessment, Experience sampling, Health care, Psychology, Psychology

## Abstract

What are you feeling *right now?* Were you aware of where your mind was a moment ago*?* Questions like these provide simple, face valid measures of momentary experience. Ultra-brief measures using the experience sampling method (ESM), consisting of a few items that can be completed in about a minute, may also be highly scalable and increase engagement in applied contexts. We developed single-item measures of teen wellbeing using ESM in four domains: awareness, connection, insight, and purpose. Then, we evaluated item relevance and clarity through user testing with 12 teens aged 14–18 years old and made revisions based on their feedback. Finally, we tested the new items in the context of ESM with 156 teens aged 13–18 years old, over a period of 8 days with ESM questions sent via text message three times each day outside school hours. We found one or more items for each domain with acceptable validity, response variability, and a normal response distribution. We assessed convergent, divergent, and predictive validity, and found significant relationships for comparison measures of each type. Future research should further investigate using these measures in the context of interventions that include training in these skills-based domains.

## Introduction

Adolescence is a period of heightened risk for developing mental health issues^1–3^, and also a window of neurodevelopmental plasticity that provides potential for learning healthy habits and coping strategies to bolster lifelong wellbeing^4–6^. Assessment is critical to identify changes in vulnerability and opportunities to bolster adolescent mental health, as well as to evaluate effects of interventions. Moreover, given the ubiquity of technology in daily life, there is a growing need to understand teen wellbeing with measures that have potential for integration alongside technology that may impact wellbeing. Recent research has highlighted the unique vulnerability of adolescents to potential harms of social media use and a nuanced picture of the broader impact of screen time^7–9^, further motivating the need for flexible, real-time wellbeing measures. Concerns that digital devices are contributing to a broad-scale mental health crisis among adolescents^10,11^ are counterbalanced by other data showing improved outcomes, including school drop-out and delinquency^12^. Real-time measures of wellbeing, alongside mental health measures may help parse the relationships between digital technology use, prevalence of mental health disorders, and adaptive coping through self-awareness and vocalization. Mobile devices and applications can provide a means to deliver wellbeing assessments that are highly scalable, as 95% of American adolescents have access to a smartphone^13^. Some estimates indicate that adolescents use smartphones for an average of 7 h per day^14^, providing the potential for dense sampling with measures collected throughout their daily lives.

The experience sampling method (ESM) provides a means to probe momentary experience as it is occurring in real-world contexts. Using ESM to repeatedly assess experience addresses the potential biases of the global, retrospective judgements inherent to many self-report measures^15^, and can also reveal dynamic patterns across time. Accordingly, ESM measures have been shown to demonstrate incremental validity in predicting outcomes above and beyond retrospective self-report measures of the same construct^16^.

In contrast to the growing scientific literature on ESM measures of mood, stress, and resilience (i.e., recovery from stress)^17–20^, there is a relative paucity of psychological wellbeing from a eudaimonic perspective. Thus, we sought to develop novel ESM measures of eudaimonic wellbeing among adolescents, defined as a state that allows individual flourishing in which an individual is fully functioning and has realized their potential^21,22^. This conceptualization of wellbeing is not synonymous with momentary emotions, moods, or satisfaction, i.e., hedonic wellbeing^22^. It is possible to have high wellbeing while experiencing a state of stress or negative affect, or conversely, to have low wellbeing while feeling reward or satisfaction. For example, behaviors like eating junk food, prolonged scrolling through social media apps, or gambling might feel good in the moment, though would not be characterized as contributing to, nor characterizing, wellbeing. Cross-cultural research has identified similarities across seven global regions indicating distinct aspects of eudaimonic wellbeing from life satisfaction or hedonic wellbeing^23^.

The ESM measures of wellbeing developed here were based on a four domains from a recently validated self-report measure of eudaimonic wellbeing in teens: awareness, connection, insight, and purpose^24^. This self-report measure was developed from an evidence-based framework that defines facets of wellbeing that can be cultivated through training^25^, rather than symptoms of ill-being that are commonly the focus in measures of mental health. According to this theoretical framework, wellbeing can be cultivated through integrating practices and applying skills from each of these four domains into daily life^25^. Initial evidence supports this proposition: large randomized trials of the Healthy Minds Program app-targeting these skills for training through daily practices-show significant improvements in psychological distress and wellbeing^26,27^. Assessing awareness and insight, in addition to connection and purpose, distinguishes this measure, the Healthy Minds Index^24^, from other validated multi-dimensional measures of eudaimonic well-being^28^.

The four domains of wellbeing defined by this framework reflect related and interacting, yet distinct constructs that are supported by partially separable brain networks^25^. The first domain is awareness, defined as “a heightened and flexible attentiveness” to both external and internal cues^25^. In this framework, awareness includes skills like present-moment awareness, sustained attention, and focus. The domain of connection is defined as a sense of care “that promotes supportive relationships and caring interactions”^25^. Skills in the connection domain include compassion, positive person construal, and empathy. The third domain is insight, defined as self-knowledge about how feelings, thoughts, and beliefs shape subjective experience and sense of self^25^. Insight includes skills such as self-inquiry and meta-cognitive awareness, i.e., thinking about thinking. The domains of awareness and insight are most closely related in this framework, though were found to be distinct factors in prior work validating the self-report measure^24^. Finally, purpose is defined in this framework as “a sense of clarity concerning personally meaningful aims and values that one is able to apply in daily life”^25^. The domain of purpose includes skills like clarifying ones’ values, goal-setting, and behavior-value alignment. Skills across all four domains are present to various degrees across individuals and can be enhanced or diminished through development and experience, or occur spontaneously, e.g., as a moment of insight or connection^25,29–31^. The scientific literature also provides evidence that skills across all four domains can be further strengthened through training, as outlined in prior work defining this wellbeing framework^25^. Outside the context of training, these domains of wellbeing may vary both between and within individuals, depending on the individuals’ predisposition to wellbeing in these areas and the degree to which their day-to-day behaviors and practices support awareness, connection, insight, and purpose.

We sought to develop a single-item ESM measure to assess in-the-moment eudaimonic wellbeing for each of the four skills-based domains outlined by the evidence-based framework, described above (awareness, connection, insight, and purpose). An ultra-brief ESM measure reduces burden in real-world contexts, complements retrospective self-report, and has the potential to inform strategies to improve wellbeing and assess the immediate impacts of intervention strategies at scale. Prior research has demonstrated the validity of single-item measurement strategies in ESM paradigms, including predictive validity^32^. We initially developed multiple items for each of the four domains with the goal to optimize the items following feedback from user testing, test the validity of each item in an ESM context, and create a final set with one item per wellbeing domain. Creating an ESM set with one item per wellbeing domain minimizes participant burden in ESM research (and optimizes engagement) by keeping the survey brief, while maintaining a measure across all four domains. For each item, we separately examined descriptive statistics and response distributions, convergent validity with retrospective self-report measures, predictive validity with general wellbeing measures collected after the ESM period, within-person response variability, and divergent validity with concurrent stress reports and cross-domain comparisons. We hypothesized that each item would be significantly positively related to domain-specific comparison measures, and to more distal measures of general wellbeing and life satisfaction. We hypothesized that each item would be negatively associated with measures of stress-related measures, and either unrelated or very weakly related to concurrent measures of other wellbeing constructs.

## Results

### User testing

We developed a set of ESM items for each of the four evidence-based domains of wellbeing that can be trained through practice, as outlined by Dahl et al.: awareness, connection, insight, and purpose^25^. Following item development, we conducted user testing to assess whether; (1) teens understood all the ESM questions, as intended; (2) there were any red flags or language changes needed to either the questions or the response options to improve clarity and comprehension.

Overall, teens reported that the ESM items were clear and easy to understand. The qualitative feedback indicated that some of the questions were less applicable when participants were in a very calm mood, e.g., “how intense is the emotion you are feeling right now?”. One item was unclear, and participants reported experiencing it as “insulting” by implying that they were “bad” or “selfish” (“Today how often did you feel it was important to look out for one another vs. yourself?”). Thus, we dropped this item.

A few items were revised to improve clarity through more concise language. For example, one question used the word “how” three times and was revised to reduce the redundancy, and the overall length of the question. For purpose items, the word “fulfillment” resonated with teens, while for insight items, we identified items with more concrete language that were preferred over items with less concrete language, e.g. “emotional state” instead of “state of mind”. We applied the revisions to subsequent studies. A full list of initial items and corresponding revisions is provided in the Appendix.

### ESM items for psychometric evaluation

Based on the user testing, we revised the language and narrowed the ESM items to a set of 16 items for further psychometric testing (Table 1), including two context-setting questions that were present in the original set of questions, and were asked prior to awareness items 1 and 2: “What is the strongest emotion you have been feeling in the past 10 min?” and “How intense was the emotion that you were feeling in the past 10 min?”, respectively.

**Table 1:** Final list of ESM items revised from user testing.

	Item*	Mean (SD)	ICC
A	1. How much did you notice how you were feeling emotionally before we asked?**	3.2 (1.3)	0.30
	2. How aware were you of how you were feeling before we asked?***	2.6 (1.4)	0.25
	3. How distracted were you in the past 10 min? [reverse]	3.2 (1.4)	0.35
	4. Had you noticed you were feeling this way before we asked?	2.6 (1.4)	0.38
C	1. How connected to others did you feel in the past 10 min?	2.6 (1.3)	0.22
	2. In the past 10 min, how often did you feel you were supported by others?	2.6 (1.2)	0.34
	3. In the past 10 min, how often did you feel connected to others?	2.6 (1.3)	0.28
I	1. In the past 10 min, did you recognize how your feelings were influencing your outlook on things?	2.5 (1.3)	0.21
	2. In the past 10 min, did you understand why you think and feel the way you do?	3.4 (1.3)	0.31
	3. Did you recognize how your thoughts and feelings were affecting your behavior before we asked?	2.6 (1.3)	0.29
P	1. To what extent do you view what you’re doing in the past 10 min as personally fulfilling?	2.9 (1.3)	0.22
	2. How much fulfillment did you feel in the past 10 min?	2.8 (1.3)	0.29
	3. Do you see your personal values reflected in the activity you’ve been doing these past 10 min?	2.7 (1.3)	0.28
	4. How do you view what you’ve been doing in the past 10 min?	3.1 (1.2)	0.19
S	In the past 10 min, how much did you feel nervous or stressed?	3.9 (1.2)	0.18

SD, Standard deviation; ICC, Intra-class correlation as indicator of within-item variance over the sampling period, within-person; A, Awareness; C, Connection; I, Insight; P, Purpose; S, Single-item stress comparison ESM.

*Items rated on 5-point Likert scale, where 1 is least and 5 is most.

**Item A-1 was preceded by the context question, “What is the strongest emotion you have been feeling in the past 10 min?”.

***Item A-2 was preceded by the context question, “How intense was the emotion you were feeling in the past 10 min?”.

### ESM item validation study

We conducted a conventional ESM study during which participants received text messages each day for 8 days. This design allowed us to test the validity of the ESM items as assessing state fluctuations in dimensions of wellbeing during everyday life.

Participants responded to 77% of ESM items (92.4 out of 120), on average, and the mean response time per ESM set was 1.0 min. The study required participants to complete a minimum of half of the ESM surveys, and 93% of participants complied. There were 82% of participants who completed at least 70% of the ESM surveys (or 84 out of 120).

### Descriptive statistics

**Fig. 1:**
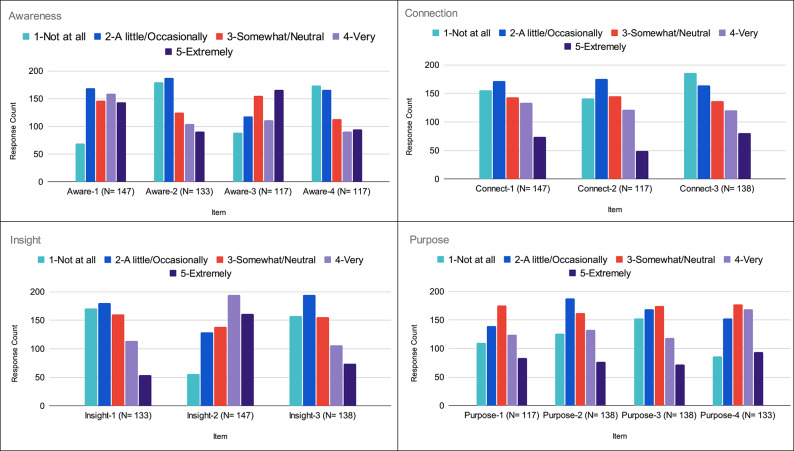
ESM item response distributions for wellbeing items. Response options were prefixed by the stems “Not at all” to “Extremely”. Sample sizes ranged from 115 to 147 and are listed along the horizontal axis for each item.

**Fig. 2:**
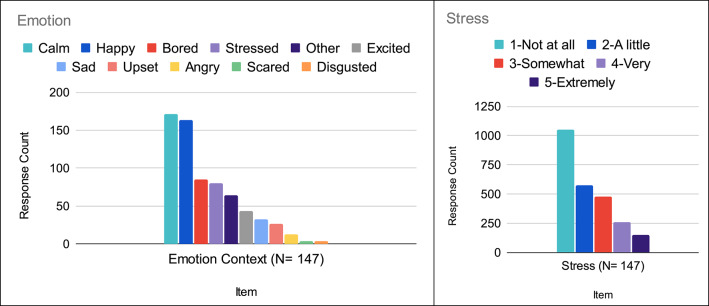
ESM item response distributions for emotion and stress items. The emotion context question preceded awareness item 1, which referenced the response to this item. The stress ESM item was included in all ESM item sets for comparison to the wellbeing ESM items and had higher overall response counts than other items. All 147 participants completed at least one emotion context question and at least one stress item. The stress ESM item was rated on a 1 to 5 Likert scale, using a similar format to the other ESM items.

The response distributions were normal for most items across domains, except for signs of possible floor effects for the following items: awareness items two and four, connection item three, and insight item one (Fig. 1). The response distribution for the single-item stress question, however, was strongly skewed, with most participants responding with very low stress levels, on average (Fig. 2). Responses to the context question that preceded awareness item 1 showed that participants on average reported feeling “calm” (24.9% of responses) or “happy” (23.8% of responses) most of the time, when queried (Fig. 2). The next most common responses were “bored” (12.4%) and “stressed” (11.7%).

**Fig. 3:**
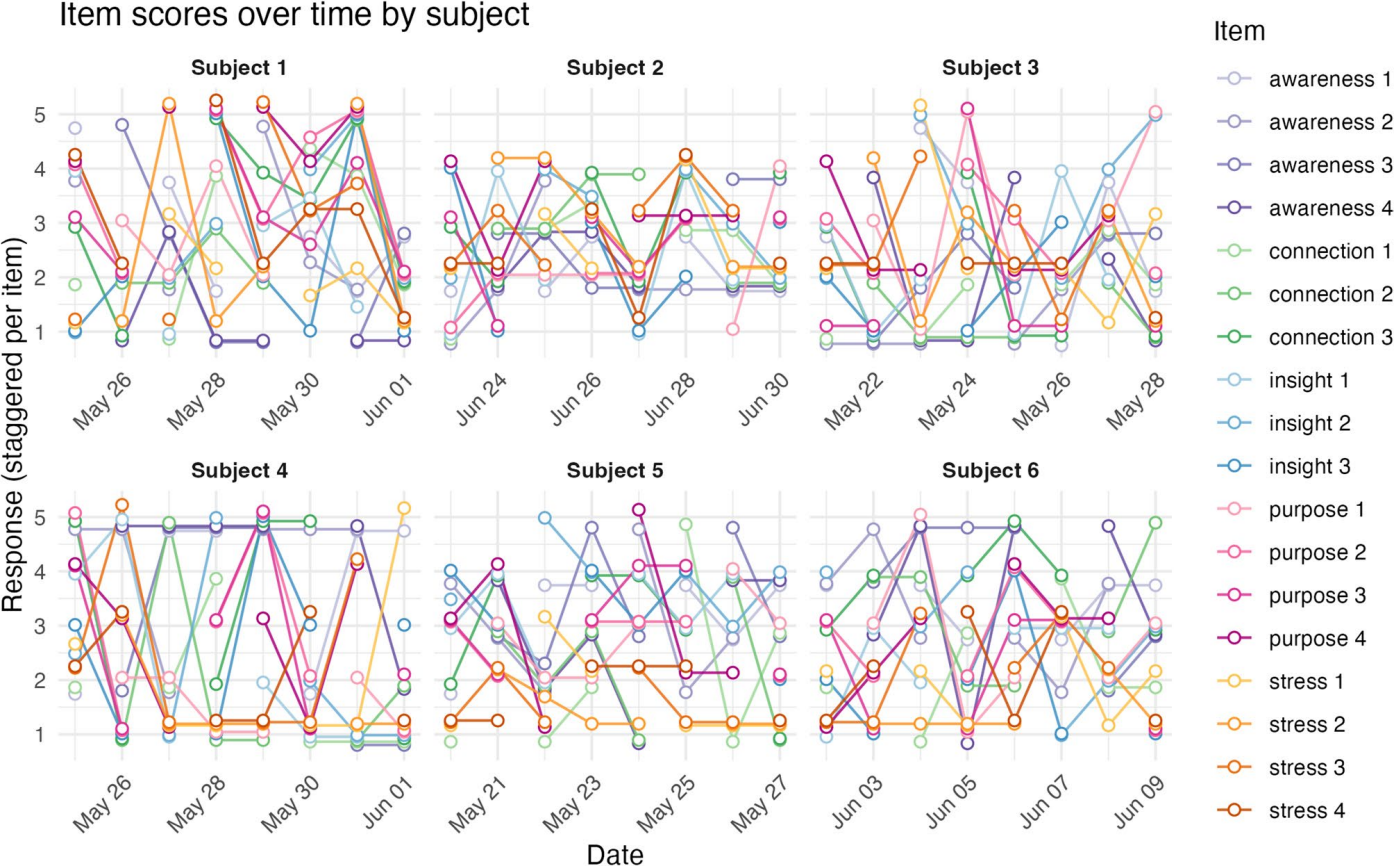
Line plots for response to all ESM items by day for six example participants. Points and lines are offset to allow visualization of overlap.

**Fig. 4:**
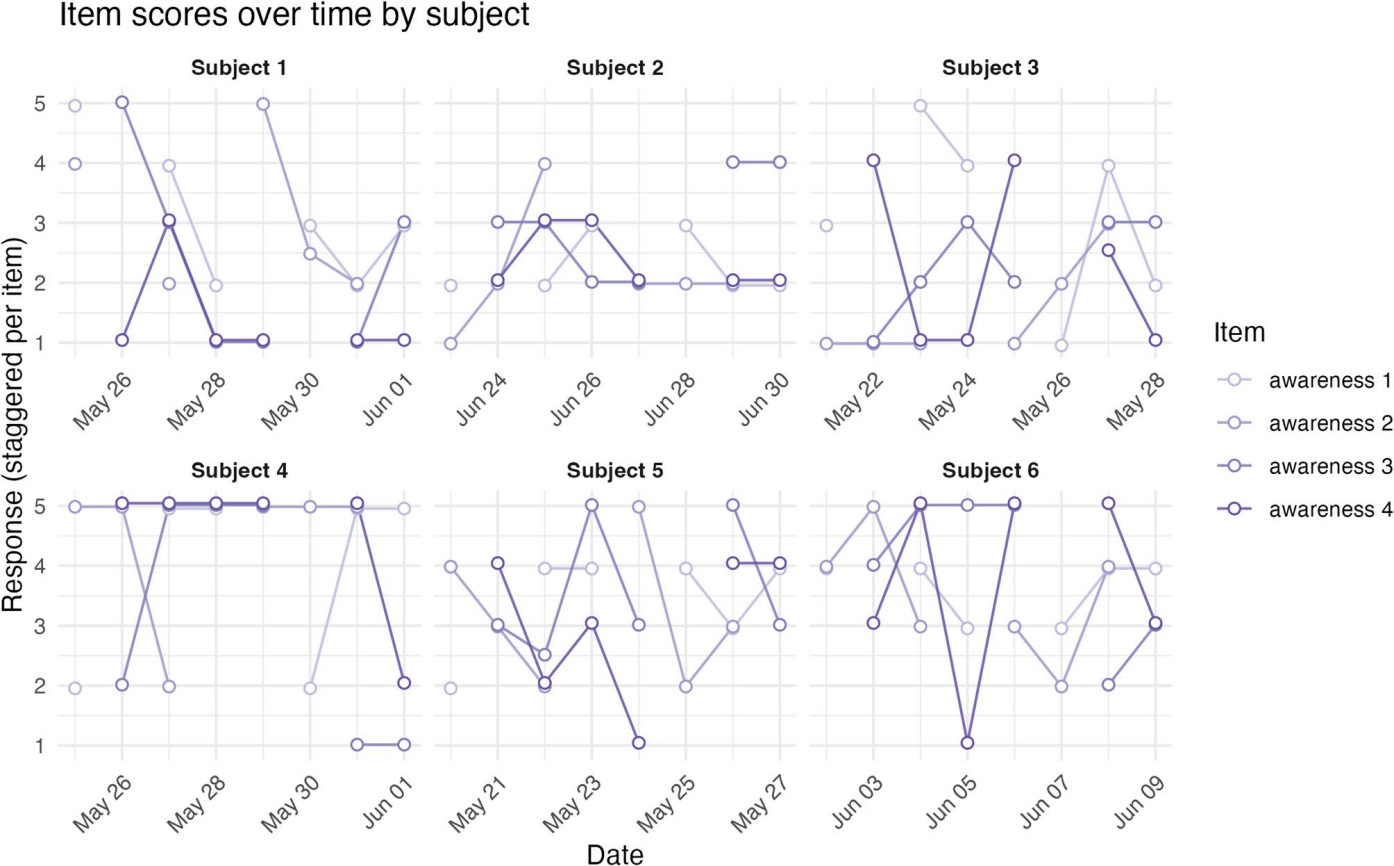
Line plots for response to ESM awareness items by day for six example participants. Points and lines are offset to allow visualization of overlap.

The between-subject variation in item ratings, as assessed by the standard deviation, ranged from 1.2 to 1.4. The within-subject variation in item ratings, as assessed by the ICC of the mixed model intercept, was relatively high, as expected (average = 0.27; range = 0.18 to 0.38). Descriptive statistics for each item are shown in Table 1. Example line plots to visualize response variability for a subset of six participants are shown in Fig. 3 for all items, and for awareness items alone in Fig. 4.

### Validity

One or more ESM item in each domain was significantly associated in the expected directions with one or more measures of general wellbeing and mood, and with two or more domain-specific comparison measures (Tables 2, 3, 4, 5). Results indicated that some items had better convergent validity than others, and best-performing items regarding convergent validity and response distribution are indicated with an asterisk in Tables 2, 3, 4, 5. The awareness items all correlated significantly with the survey measure of awareness of internal experiences and attention (ESQ), while there were no relationships between the awareness ESM items and the acting with awareness scale. Connection item 1 (“How connected do you feel in the past 10 min?”) was the only connection item that was significantly related to the survey measures of compassion and positive relations with others. For the insight domain, we found significant associations for two insight-related measures: awareness of internal experiences and reappraisal. None of the ESM items related to the survey measure of “relativity of thoughts” (CHIME). Three of four purpose ESM items had significant associations with the survey measures of purpose and meaning, and with multiple survey measures of wellbeing and positive affect. The most consistent results were for purpose item 4 (“How do you view what you’ve been doing in the past 10 min?” [from meaningless to meaningful]), while purpose item 3 (“Do you see your personal values reflected in the activity you’ve been doing these past 10 min?”) showed poor convergent validity.

**Table 2:** Relationships between ESM awareness items and comparison measures.

Measure	ESM item											
	Item 1: How much did you notice how you were feeling emotionally before we asked?*			Item 2: How aware were you of how you were feeling before we asked?			Item 3: How distracted were you in the past 10 min?			Item 4: Had you noticed you were feeling this way before we asked?		
	*β*	R^2^	*P*	*β*	R^2^	*P*	*β*	R^2^	*P*	*β*	R^2^	*P*
Life Sat	**.162**	**.03**	**.040**	.147	.02	.080	.060	.01	.780	.139	.03	.120
HM Index	**.319**	**.12**	** < .001**	**.240**	**.06**	** < .001**	.108	.02	.440	**.292**	**.11**	** < .001**
WHO-5	**.126**	**.02**	**.040**	.020	< 0.01	.700	.057	< .01	.773	.126	.02	.053
PANAS-PA	**.163**	**.03**	**.040**	.105	.012	.267	.002	< .01	.980	.182	.04	.053
PANAS-NA	− .072	.03	.331	− .071	.01	.387	.016	< .01	.937	− .091	.01	.280
STAI–state	.099	< .01	.048	.019	< 0.01	.700	.014	< .01	.937	.044	< .01	.446
PSS	.044	< .01	.370	− .069	< 0.01	.320	− .037	< .01	.784	− .035	< .01	.510
1-item stress	− .097	.01	.200	− .065	.01	.387	− .118	.02	.440	− .102	.01	.240
CHIME-A Act	− .003	< 0.01	.961	.045	< 0.01	.479	.071	.02	.106	.031	< .01	.680
CHIME-A Aware	**.290**	**.10**	** < .001**	**.232**	**.06**	** < .001**	.102	.01	.171	**.223**	**.06**	**.005**
ESQ	**.167**	**.03**	**.022**	**.150**	**.03**	**.021**	.143	.03	.106	**.233**	**.07**	**.004**

Significant values are in [bold].

*Indicates the best-performing item within the domain; *β*, Standardized regression coefficient (beta); Sat. = satisfaction; HM = Healthy Minds Index well-being; WHO-5 = World Health Organization 5-item well-being index; PANAS = Positive and Negative Affect Scale; PA = positive affect; NA = negative affect; STAI = State-Trait Anxiety Inventory – State scale; PSS = Perceived Stress Scale; CHIME-A = Comprehensive Inventory of Mindfulness Experiences –Adolescents; Act. = acting with awareness; Aware = awareness of internal experiences; ESQ = Emotional Styles Questionnaire attention scale; P-values FDR-corrected for multiple comparisons.

**Table 3:** Relationships between ESM connection items and comparison measures.

Comparison measure	ESM item								
	Item 1: How connected to others did you feel in the past 10 min?*			Item 2: In the past 10 min, how often did you feel you were supported by others?			Item 3: In the past 10 min, how often did you feel connected to others?		
	*β*	R^2^	*P*	*β*	R^2^	*P*	*β*	R^2^	*P*
Life Sat	**.150**	**.03**	**.040**	**.183**	**.04**	**.040**	**.197**	**.04**	** < .001**
HM Index	**.206**	**.05**	** < .001**	**.255**	**.08**	** < .001**	**.242**	**.07**	** < .001**
WHO-5	**.141**	**.02**	** < .001**	.095	.01	.160	**.139**	**.02**	** < .001**
PANAS-PA	.128	.02	.053	.146	.03	.107	**.197**	**.04**	** < .001**
PANAS-NA	− .063	.01	.300	− .089	.01	.267	− .085	.01	.217
STAI-state	**.105**	**.01**	**.040**	.052	< .01	.354	.061	< .01	.217
PSS	.060	.01	.229	.003	< .01	.950	.002	< .01	.970
1-item stress	− .130	.02	.048	− .102	.01	.208	− .101	.01	.160
Pos. Rel	**.108**	**.01**	**.021**	.085	.01	.202	.105	.01	.210
Compassion	**.158**	**.03**	**.021**	.086	.01	.239	.011	< .01	.868

Significant values are in [bold].

*Indicates the best-performing item within the domain; Sat., Satisfaction; HM, Healthy Minds Index well-being; WHO-5, World Health Organization 5-item well-being index; PANAS, Positive and Negative Affect Scale; PA, Positive affect; NA, Negative affect; STAI, State-Trait Anxiety Inventory—State scale; PSS, Perceived Stress Scale; Pos. Rel., Positive relations with others; *P*-values FDR-corrected for multiple comparisons.

**Table 4:** Relationships between ESM insight items and comparison measures.

Comparison measure	ESM item								
	Item 1: In the past 10 min, did you recognize how your feelings were influencing your outlook on things?*			Item 2: In the past 10 min, did you understand why you think and feel the way you do?			Item 3: Did you recognize how your thoughts and feelings were influencing your behavior before we asked?		
	*β*	R^2^	*P*	*β*	R^2^	*P*	*β*	R^2^	*P*
Life Satisfaction	.139	.02	.120	.087	.01	.267	.084	.01	.377
HM index	**.261**	**.07**	** < .001**	**.281**	**.10**	** < .001**	**.302**	**.10**	** < .001**
WHO-5	.043	< .01	.537	.108	.01	.120	.053	< .01	.377
PANAS-PA	.121	.02	.187	.039	< .01	.570	**.189**	**.04**	**.040**
PANAS-NA	− .047	< .01	.537	− .092	.01	.267	− .146	.03	.080
STAI-state	− .008	< .01	.319	.060	< .01	.267	.004	< .01	.940
PSS	− .044	< .01	.537	.028	< .01	.570	− .048	< .01	.377
1-item stress	− .046	< .01	.537	− .106	.01	.267	− .100	.01	.280
CHIME-A Awa	**.205**	**.05**	**.002**	**.161**	**.03**	**.031**	**.264**	**.08**	** < .001**
CHIME-A Rel	.116	.02	.083	.009	.01	.891	.138	.02	.053
Reappraisal	**.231**	**.06**	**.001**	**.152**	**.03**	**.031**	.138	.02	.053

Significant values are in [bold].

*Indicates the best-performing item within the domain; HM, Healthy Minds; WHO-5, World Health Organization 5-item well-being index; PANAS, Positive and Negative Affect Scale; PA, Positive affect; NA, Negative affect; STAI, State-Trait Anxiety Inventory—State scale; PSS, Perceived Stress Scale; CHIME-A, Comprehensive Inventory of Mindfulness Experiences—Adolescents; Aware, Awareness of internal experiences; Rel., Relativity of thoughts; *P*-values FDR-corrected for multiple comparisons.

**Table 5:** Relationships between ESM purpose items and comparison measures.

Measure	ESM item											
	Item 1: To what extent do you view what you’re doing in the past 10 min as personally fulfilling?			Item 2: How much fulfillment did you feel in the past 10 min?			Item 3: Do you see your personal values reflected in the activity you’ve been doing these past 10 min?			Item 4: How do you view what you’ve been doing in the past 10 min?*		
	*β*	R^2^	*P*	*β*	R^2^	*P*	*β*	R^2^	*P*	*β*	R^2^	*P*
Life Sat	.107	.01	.180	**.195**	**.04**	** < .001**	.085	.01	.336	**.165**	**.03**	**.026**
HM index	**.218**	**.05**	** < .001**	**.230**	**.06**	** < .001**	.148	.03	.080	**.173**	**.03**	** < .001**
WHO-5	.086	.01	.180	**.134**	**.02**	**.016**	− .070	.01	.320	.107	.01	.060
PANAS PA	**.151**	**.03**	**.040**	**.211**	**.05**	** < .001**	.158	.03	.080	**.148**	**.02**	**.027**
PANAS NA	− .057	< .01	.480	− .076	.01	.320	− .064	.01	.427	− .092	.01	.192
STAI-state	.025	< .01	.697	.035	< .01	.526	.017	< .01	.834	.028	< .01	.550
PSS	− .019	< .01	.710	− .018	< .01	.710	.007	< .01	.880	− .047	< .01	.377
1-item stress	− .053	< .01	.480	− **.158**	**.03**	**.016**	− .105	.01	.267	− .082	.01	.213
Costin	**.143**	**.02**	**.016**	**.216**	**.05**	**.002**	.121	.02	.128	**.155**	**.03**	**.009**
Francis	**.146**	**.02**	**.016**	**.186**	**.04**	**.005**	.076	.01	.255	**.181**	**.04**	**.005**

Significant values are in [bold].

*Indicates the best-performing item within the domain; Sat., Satisfaction; HM, Healthy Minds Index well-being; WHO-5, World Health Organization 5-item well-being index; PANAS, Positive and Negative Affect Scale; PA, positive affect; NA, negative affect; STAI, State-Trait Anxiety Inventory—State scale; PSS, Perceived Stress Scale; *P*-values FDR-corrected for multiple comparisons.

Conversely, there were no significant associations between the stress ESM question and measures of general wellbeing, and only significant relationships with negative affect and the single-item stress measure, reflecting a general indicator of distress (Table 6). The stress ESM measure was associated with 2 of the 9 domain-specific measures: attention and awareness of internal experiences.

**Table 6:** Relationships between stress ESM item and comparison measures.

Measure	*β*	R^2^	*P*
Life satisfaction	− .113	.02	.08
Healthy minds index	− .077	.01	.16
World Health Organization 5-item well-being index	− .072	.01	.08
Positive and negative affect scale-positive affect	− .013	< .01	.80
Positive and negative affect scale-negative affect	**.170**	**.04**	** < .01**
State trait anxiety inventory-state	− .021	< .01	.61
Perceived stress scale	.038	< .01	.36
1-Item stress	**.215**	**.06**	** < .01**
CHIME-A acting with awareness	− **.140**	**.02**	**.040**
CHIME-A awareness of internal experiences	− .063	.01	.458
Emotional styles questionnaire attention	− **.127**	**.02**	**.040**
Positive relations with others	− .035	< .01	.458
Compassion	.010	< .01	.957
CHIME-A relativity of thoughts	.001	< .01	.979
Emotion regulation questionnaire-reappraisal	.022	< .01	.874
Costin purpose	− .058	< .01	.256
Francis 1-item purpose	− .122	.02	.458

Significant values are in [bold].

CHIME-A, Comprehensive inventory of mindfulness experiences-adolescents; *P*-values FDR-corrected for multiple comparisons.

### Within-person validity

There were significant within-person associations between the stress ESM item and all four awareness ESM items and two of the three connection ESM items (Table 7). As expected, connection items were inversely associated with stress, such that higher stress related to lower wellbeing. Conversely, awareness items were positively associated with stress.

**Table 7:** Relationships between concurrent ESM wellbeing items and ESM stress.

Wellbeing item	*β*	CI	*P*
Awareness 1	.157	[0.50, .262]	.004
Awareness 2	.185	[.038, .328]	.010
Awareness 3	.23	[.147, .317]	< .001
Awareness 4	.17	[.080, .261]	< .001
Connection 1	− .114	[− .222, − .007]	.039
Connection 2	− .059	[-.147, .030]	.196
Connection 3	− .145	[− .267, − .023]	.021
Insight 1	.005	[− .098, .107]	.930
Insight 2	− .047	[− .199, .106]	.550
Insight 3	.004	[− .108, .116]	.942
Purpose 1	− .045	[− .127, .038]	.290
Purpose 2	− .058	[− .222, .105]	.486
Purpose 3	.057	[− .065, .179]	.359
Purpose 4	− .063	[− .172, .045]	.253

ESM, Experience sampling method; *β*, standardized regression coefficient (beta); CI, confidence interval.

We tested discriminant validity by examining relationships between wellbeing ESM items that were assessed concurrently (e.g., within the same set), as they corresponded to distinct constructs. The best-performing ESM items from each domain (in terms of convergent and predictive validity, response distributions) were weakly or unrelated to items from other ESM domains. (Table 8). For example, there was no relationship between insight item one and the concurrently collected items for connection or awareness; nor for purpose item four and concurrent measures of insight and connection (*p* > .050; corresponding to the best-performing wellbeing items in these domains). Awareness item one and connection item one were weakly, though significantly, related (standardized beta = .128, *p *= 0.012), with a similar effect size to the awareness and connection item association with general wellbeing indexed by the WHO-5 (standardized betas = .126 and .141, respectively). There were significant relationships between items that were poorly performing on other tests and measures of constructs that were hypothesized to be distinct. For example, connection item 3 was significantly associated with concurrent ESM measures of insight (*p* = .002) and purpose (*p* < .001), while unrelated to convergent measures of compassion and positive relation with others. (Table 8).

**Table 8:** Relationships between concurrent ESM wellbeing items.

Item comparison	Covariates	*β*	CI	*P*
A1 versus C1	Stress, I1	.128	[.025, .233]	.012
A1 versus I1	Stress, C2	.051	[− .043, .147]	.283
C1 versus I1	Stress, A1	.086	[− .005, .178]	.062
A2 versus I2	Stress, P2	.253	[.120, .385]	< .001
A2 versus P2	Stress, I2	.066	[− .075, .208]	.359
I2 versus P2	Stress, A2	.280	[.151, .410]	< .001
A3 versus A4	Stress, C2, P1	.041	[− .039, .121]	.317
A3 versus C2	Stress, A4, P1	− .007	[− .089, .074]	.861
A3 versus P1	Stress, A4, C2	− .063	[− .138, .013]	.105
A4 versus C2	Stress, A3, P1	− .043	[− .121, .045]	.289
A4 versus P1	Stress, A3, C2	.089	[.024, .176]	.018
C2 versus P1	Stress, A3, A4	.341	[.272, .410]	< .001
C3 versus I3	Stress, P3, P4	.133	[.049, .217]	.002
C3 versus P3	Stress, I3, P4	.412	[.328, .496]	< .001
C3 versus P4	Stress, I3, P3	.076	[− .006, .158]	.072
I3 versus P3	Stress, C3, P4	.148	[.047, .250]	.004
I3 versus P4	Stress, C3, P3	.080	[− .010, .171]	.082
P3 versus P4	Stress, C3, I3	.138	[.056, .221]	.001

ESM, experience sampling method; A, awareness; C, connection; I, insight; P, purpose; First item listed in Item Comparison was set as the dependent variable in a linear model including the Covariates listed; *β*, standardized regression coefficient (beta); CI, confidence interval.

## Discussion

The present study introduces a novel set of ESM items with validation using a longitudinal study design with mini surveys collected three times daily. We created and tested the new set of ESM items to measure skills-based aspects of wellbeing, which were loosely derived from the recently published Healthy Minds Index of wellbeing^24^. Following expert consultation and user testing, we narrowed the ESM items to 4 each in the domains of awareness and purpose, and 3 each for the domains of connection and insight.

We assessed the validity of the final 14 questions in an ESM texting study. Responses generally had normal distributions for at least one or more items in each of the four wellbeing domains, whereas the single-item stress comparison measure was highly skewed right, exhibiting a strong floor effect. We found evidence for convergent validity for at least one of our ESM items for each of the four domains, given the significant correlations with domain-related measures. Awareness item one (“How much did you notice how you were feeling emotionally before we asked?” following the context question, “What is the strongest emotion you have been feeling in the past 10 min?”) had significant, positive associations with the hypothesized convergent measures of attention and awareness of internal experiences, with medium and small effect sizes, respectively. None of the awareness items were related to the measure of “acting with awareness” (CHIME). Retrospectively, this is consistent with the content of the awareness ESM items reflecting awareness to thoughts and feelings, more so than awareness in relation to behavior. Connection item one (“How connected to others did you feel in the past 10 min?”) had significant, positive associations with hypothesized convergent measures of compassion and positive relations with others, at a small effect size. Insight item one (“In the past 10 min, did you recognize how your feelings were influencing your outlook on things?”) was significantly, positively associated with hypothesized convergent measures of awareness of internal experiences and reappraisal with small-medium effect sizes. None of the insight items were associated with the hypothesized convergent measure of awareness of thoughts’ relativity. Purpose item four (“How do you view what you’ve been doing in the past 10 min?” [from meaningless to meaningful]) was significantly and positively associated with both hypothesized convergent measures of purpose in life, with small-medium effect sizes. There were also one or more items tested in each domain where we did not see significant associations with any or all hypothesized convergent measures, and we have highlighted the best-performing items that we would recommend for future research.

The ESM items significantly correlated to all four distal measures of wellbeing and had mostly non-significant relationships with measures of general distress, such as stress, anxiety, and negative affect. The best-performing ESM items in each domain had significant, positive associations with hypothesized predictive comparison measures of positive affect and satisfaction with life, which may reflect the greater utility of these items as measures of flourishing. Awareness item one was positively associated with all four distal wellbeing measures: life satisfaction, positive affect, WHO-5 wellbeing, and Healthy Minds Index wellbeing. Connection item one was not associated with positive affect, purpose item four was unrelated to WHO-5 wellbeing, and insight item one was only associated with the closely related Healthy Minds Index wellbeing outcome. Alternate items in each domain performed similarly or better in relation to these more global wellbeing outcomes but failed to meet convergent validity for domain-related convergent measures and are thus not recommended as valid measures for the given domain. Conversely, the single-item stress ESM measure was significantly related to 2 of 4 stress-related trait measures, and only one of the three wellbeing measures. The effect sizes in all cases were relatively weak, likely due to making comparisons between different self-report modalities (i.e., retrospective versus present-moment experience sampling). Overall, the new wellbeing items developed herein are best suited to measure aspects of flourishing and the relevant domain-specific construct (i.e., awareness, connection, insight, or purpose), while these items don’t well represent stress, anxiety, or negative affect. Standard stress ESM items, such as the control item used in this study, are much better suited to capture those negative, stress-related constructs.

Within-person associations between the ESM wellbeing items and concurrent stress showed significant associations for items within the awareness and connection domains, while controlling for responses to concurrent ESM items. Interestingly, there were variations in the direction of associations between responses to wellbeing ESM items and stress ratings, across domains. Responses to items in the awareness domain were higher with higher levels of in-the-moment stress, which could reflect aspects of the higher arousal state of the stress experience bringing greater awareness to present moment experience. In retrospect, these results are consistent with the way in which the stress response recruits attentional systems to respond to environmental stimuli and increase vigilance to potential^33–35^. Conversely, higher responses for items in the connection domain were associated with lower stress in the moment, demonstrating discriminant validity of these items. We expected negative relationships between in-the-moment stress and wellbeing items, given the hypothesis that wellbeing and stress are inversely correlated, in general. However, there was no relationship between in-the-moment stress and concurrent insight or purpose, when controlling for items in the other domains. These results, in part, lend further evidence to the non-specificity of the stress measure in relation to skills-based domains of wellbeing, and rather as a marker for general distress.

While we were unable to compare intercorrelations for items within the same domain, as they were not included in concurrent question sets, we examined relationships across different domains as a first step in testing discriminant validity. Results indicated evidence for discriminant validity of the best-performing ESM item for each of the wellbeing domains, which was weakly or unrelated to concurrent ESM items for other domains (i.e., constructs). Conversely, wellbeing items that performed less well for convergent and predictive validity, also failed to show discriminant validity. In this way, the best performing ESM items demonstrated discriminant, convergent, and predictive validity, while the poorly performing items were systematically lacking in one or more validity tests. We recommend item one from awareness, connection, and insight domains, and item four from the purpose domain as valid options for future ESM research on these wellbeing domains.

The present study was limited to teen participants residing within the United States of America (U.S.) who were able to read in English, who had an internet-enabled smartphone, and participated in the online research platform, Qualtrics. Participants identified as 62.2% female, 28.9% male, 7.7% non-binary, and 1.3% other gender identity or declined to respond. Race and ethnicity data indicated that participants identified as 9.0% Aboriginal or Native American, 21.8% Black, 7.1% East Asian, 4.5% South Asian, 48.1% White, and 6.4% as other ethnicity or declined to respond. Overall, 25.0% of participants identified as Latinx. To generalize this work more broadly, future research should sample from a more diverse population, including with participants outside the U.S. and with additional recruitment methods, including adults.

The present study outlines the development of single items to assess wellbeing within the domains of awareness, connection, insight, and purpose; and provides initial evidence for item validity for the best-performing items, noted in the tables and Results. Use of single-item ESM has potential to increase engagement through reduced burden, especially for applied, non-academic contexts. Future studies should further test the validity and utility of these ESM items over longer timeframes, in adults and non-U.S. populations, and compared to commonly used ESM measures. The inclusion of validated ESM items to assess domains of teen wellbeing that are amenable to change through training has potential to further benefit efforts to promote teen flourishing. Future research is needed to examine sensitivity of these single-item ESM wellbeing measures in response to training, including with brief, ecological momentary interventions targeting each domain.

## Methods

Recruitment and data collection began in April 2021 for the user experience research and ended with the validation study in June 2022. Table 9 provides the demographic data for the participants in each study. All participants provided written assent in a digital consent form. This study was approved by the Advarra CIRB, and all research was performed in accordance with the relevant guidelines and regulations.

**Table 9:** Participant demographics for all studies.

Study		User testing	ESM item validation
Sample size		12	156
Mean age (years), SD		16.0 (1.3)	16.5 (1.3)
Gender	Female	7	97
	Male	4	45
	Nonbinary	0	12
	Other	1	2
Race	Aboriginal/Native American	1	14
	Black	2	34
	East Asian	2	11
	South Asian	0	7
	White	7	75
	Other/unknown	0	10
Ethnicity	Latinx	1	39
	Not Latinx	7	112
	Unknown	4	5

Multiple racial categories could be selected.

SD, Standard deviation; ESM, Experience sampling method.

### User testing

The initial set of ESM items included 21 questions, in addition to 2 context questions, to assess in-the-moment experience in four domains of wellbeing (awareness, connection, insight, and purpose). Items were generated based on conceptual definitions for each construct, which were operationalized according to the wellbeing framework described by Dahl, Wilson-Mendenhall & Davidson^25^. Experts helped to refine the initial items, which were then submitted for user testing. Three of the authors contributed to the initial item generation and have expertise in wellbeing and measurement (Drs. Davidson, Tatar, and Wilson-Mendenhall). In addition, Drs. Davidson and Wilson-Mendenhall were among the original authors of the theoretical framework. Three additional experts provided input on item development, including in the areas of adolescent development, measurement, and the theoretical framework (Drs. Dahl, Goldberg, and Hirshberg). Items were generated and revised asynchronously in a shared document, using comments to discuss edits, concerns, and suggestions for improvements. The full list of ESM items is included in the Appendix.

One set of ESM items was delivered via text message each day, for 4 days. The text message directed participants to click on a web link that initiated ESM items on an unmoderated user test app called Lookback. On Lookback, participants simultaneously completed the ESM items and recorded their think-aloud thoughts on video. The survey presented each of the ESM items in the set, one at a time, and they were asked to select a response. Teens were asked to read each question aloud as they took the survey, and to share (aloud) “what comes to mind when you read the question”. The user research team reviewed all the responses, and made detailed notes about the themes, recommendations, and any red flags that arose.

### ESM item validation study

#### Participants

Participants (*n* = 156) aged 13 to 18 years were recruited through an online survey platform (Qualtrics) to complete an online survey to measure wellbeing. The IRB waived the requirement for parental consent of minors, as the study was deemed no more than minimal risk to participants. Participants were compensated at the conclusion of the study, with rates based on the policies of the recruitment organization. Participants had to complete a minimum of 60% of the ESM surveys, and the final comparison survey, to qualify as completing the study for compensation. Participants received a $5 bonus if they completed 80% or more of the surveys, and a $10 bonus for completing 100% of the surveys. In addition, 5 participants were randomly selected to receive an additional $50 bonus, from among those who completed all the surveys. See Table 8 for detailed demographic information.

#### Data collection

Participants completed online surveys through Qualtrics, using TextIt to send scheduled text messages to participants, given the flexibility of this method to deliver web-based ESM surveys on a set schedule. Participants completed online assent and enrollment in the study and then initiated the texting flow by sending a text message to the study phone number. There were 120 ESM items, in total, delivered across 24 total text messages, with 5 questions per text. For 8 days, participants received three texts each day, in the morning, afternoon, and late evening. Texts were timed to occur at set times outside the hours of the school day, based on one of three morning starting times that participants selected as most convenient for their schedule. The early option had texts scheduled for 6:00 AM, 3:00 PM, and 7:00 PM. The late option had texts scheduled for 8:00 AM, 5:00 PM, and 9:00 PM. The middle option had texts scheduled for 7:00 AM, 4:00 PM, and 8:00 PM. The ESM response window closed at the time that the subsequent ESM text was initiated. The texts invited teens to complete a brief “mini survey”. They could also opt out of texting at any time. Following the 8-day texting period, participants completed a set of surveys remotely via Qualtrics. Eighty-one participants completed all surveys following the texting period (sample sizes for each measure are provided in Table 10). All survey and response data were coded and de-identified. Contact information was stored separately from study data and was deleted upon completion of data collection.

**Table 10:** Comparison surveys: descriptives and internal consistency.

Scale	Domain	Scale range	N	Mean (SD)	Alpha
Life satisfaction	Wellbeing	0–10	81	6.2 (2.1)	n/a
Healthy minds index		1–5	85	2.7 (0.7)	0.85
WHO 5-item well-being index		0–25	81	11.9 (4.4)	0.77
PANAS-positive affect		10–50	84	23.8 (9.0)	0.91
PANAS-negative affect		10–50	84	15.9 (6.4)	0.89
Perceived stress scale		0–40	81	24.4 (7.0)	0.86
State-trait anxiety inventory–state		20–80	84	42.8 (5.5)	0.92
CHIME-A acting with awareness	Awareness	1–6	82	4.6 (1.2)	0.68
CHIME-A awareness		1–6	81	4.1 (1.1)	0.81
ESQ-attention		1–7	81	3.8 (1.3)	0.72
DPES-compassion	Connection	1–7	81	5.5 (1.2)	0.89
PWB-positive relations		6–42	81	24.3 (6.1)	0.74
CHIME-A relativity of thoughts	Insight	1–6	81	4.4 (0.9)	0.70
ERQ-reappraisal		6–42	82	27.7 (7.3)	0.88
Francis 1-item purpose	Purpose	1–5	83	3.6 (1.1)	n/a
Costin purpose		1–7	83	5.0 (1.4)	0.88

SD, Standard deviation; WHO, World Health Organization; PANAS, Positive and negative affect scale; CHIME-A, Comprehensive inventory of mindfulness experiences-adolescents; ESQ, Emotional styles questionnaire; DPES, Dispositional positive emotions scale; PWB, Psychological well-being; EPOCH, Engagement, perseverance, optimism, connectedness, and happiness.

#### Measures

Participants were informed that they would receive 3 text messages each day with a link to a “mini survey”. Each text message, or ESM sampling period, prompted participants to complete one of four sets of ESM items (Table 10). Each set consisted of four of our ESM items. The awareness items were each preceded by a context question that was referenced in the subsequent ESM item (Table 1). Thus, items within a domain did not co-occur with all the other items within that same domain, given the aim to develop single-item measures. For example, awareness item 1 (“How much did you notice how you were feeling before we asked?”) was preceded by the context question: “What is the strongest emotion you have been feeling in the past 10 min?” The awareness items always came first in the ESM set, followed by items from the other domains in a random order. Each ESM item set was preceded by the following instruction: “All the questions in this mini-survey will ask you about the past 10 min—please answer them considering the past 10 min BEFORE you began this survey”. Question sets were presented in a quasi-random, balanced order, such that each set appeared twice at each time of day (morning, afternoon, or early evening) over the course of the 8-day period. Thus, participants completed each set (or item) a total of six times.

All awareness and insight items were rated on a scale from “Not at all aware” to “Extremely aware”, apart from awareness item 3, which was rated on a scale from “Extremely distracted” to “Not at all distracted”. Connection item 1 was rated on a scale from “Not at all connected” to “Extremely connected”, while connection items 2 and 3 were rated on a scale from “Not at all [supported/connected]” to “Extremely often [supported/connected]”. Purpose items 1, 2, and 3 were rated on a scale from “Not at all [fulfilling/fulfilled/reflected]” to “Extremely [fulfilling/fulfilled/reflected]” (respectively), while purpose item 4 was rated on a scale from “Extremely meaningless” to “Extremely meaningful”. A single-item stress question was also included as a comparison measure to examine whether the new ESM items performed similarly or differently with regards to the response characteristics and relationships with both general wellbeing and domain-specific measures, and to compare within-person relationships between stress and wellbeing in each domain. The stress item was adapted from item 3 of the Perceived Stress Scale^36^: “In the past 10 min, how much did you feel nervous or stressed?” The stress question was rated on a scale from “Extremely stressed” to “Not at all stressed”. See the Appendix for item sets and detailed response options.

We assessed predictive validity across all domains in comparison to life satisfaction collected after the ESM period, using the Personal Wellbeing Index^37^, the Healthy Minds Index-Wellbeing^24^, the WHO-5 Wellbeing Index^38^, and the Positive and Negative Affect Scales (PANAS)-Positive Affect^39^. We used the PANAS-Negative Affect^39^, Perceived Stress Scale^36^, and State-Trait Anxiety Inventory^40^ as comparison measures for discriminant validity across domains. For domain-specific comparison measures, we focused on convergent validity and thus expected positive associations between each domain item and the corresponding comparison measures. ESM items in the awareness domain were assessed compared to the CHIME-A Acting with Awareness and Awareness of Internal Experiences scales^41^, and the ESQ-Attention scale^42^. The DPES-Compassion scale^43^ and the PWB-Positive Relations scale^28^ were used for the connection domain; CHIME-A Relational^41^ and ERQ-Reappraisal^44^ for insight; Francis 1-item Purpose^45^ and Costin Purpose^46^ for the purpose domain. See Table 9 for response averages and variance, and internal consistency of all comparison measures.

#### Analysis

All available data were used for analysis, regardless of whether participants were missing more than 60% of the ESM responses. First, we examined the descriptive statistics for each measure, including means and standard deviations across participants, intra-class correlations (ICC) by item as a measure of within-person variability, by modeling the model intercept of ratings for each item, separately, in a mixed model, and extracted the ICC using the “performance” package (version 0.12.0) “icc” function in R Statistics^47,48^.

We tested the convergent, predictive, and discriminant validity of each ESM item, using a repeated measures mixed-effects model with the “lme4” package (version 1.1-35.3) “lmer” function in R statistics. Each comparison validation measure was separately regressed onto each ESM item, including by-person random effects. Multiple comparisons within each family of tests (e.g., for relationships with a given item and domain-related comparison measures) were corrected using False Discovery Rate (FDR), and corrected p-values are reported for all tests. Finally, we tested within-person correlations between in-the-moment stress ratings and each of the new wellbeing ESM items using the same modeling strategy, whereby responses to each set of wellbeing ESM items were regressed on responses to the stress ESM item that appeared concurrently in time (i.e., in the same set). Thus, relationships between in-the-moment stress and wellbeing items controlled for the effects of the concurrent items and reflected unique variance of each specific domain item.

## Data Availability

The datasets generated and analyzed during the current study are available on the Open Science Framework, https://osf.io/bsdz2/?view_only=f79513cf70554913b1d865b9d21d1e36.

## Appendix

### Initial item bank for user testing

Awareness items:

1. What are you feeling just now? [context question 1]
  - Revised to: What is the strongest emotion you have been feeling in the past 10 min?
2. How aware were you of how you were feeling just now?
  - Revised to: How much did you notice how you were feeling emotionally before we asked?
3. How intense was the emotion you were feeling just now? [context question 2]
  - Revised to: How intense was the emotion that you were feeling in the past 10 min?
4. In the past few moments, did you recognize how your state of mind was influencing your perspective?
  - Revised to: How aware were you of how you were feeling before we asked?
5. How focused were you just now?
  - Revised to: How distracted were you in the past 10 min?
6. How aware were you of feeling this way?
  - Revised to: Had you noticed you were feeling this way before we asked?

Insight items:

1. How aware are you right now of how your current beliefs and expectations are influencing how you are experiencing the world?
  - Revised to: In the past 10 min, did you recognize how your feelings were influencing your outlook on things?
2. In this moment, do you feel you understand why you feel and think the way you do?
  - Revised to: In the past 10 min, did you understand why you think and feel the way you do?
3. Over the past 24 h, how frequently have you noticed that your mental/emotional state was influencing your take on things?
  - Revised to: Did you recognize how your thoughts and feelings were influencing your behavior before we asked?

Connection items:

1. How do you feel right now? [response options from lonely to neutral to connected; dropped]
2. How connected do you feel with others right now?
  - Revised to: How connected to others did you feel in the past 10 min?
3. Today, how often did you notice receiving support from others?
  - Revised to: In the past 10 min, how often did you feel you were supported by others?
4. Today, how often did you feel close to others?
  - Revised to: In the past 10 min, how often did you feel connected to others?
5. Today, how often did you feel it was important to look out for one another vs. yourself? [dropped]
6. Please answer how your relationship with each of the people below was today … [response options: Terrible; Somewhat terrible; Meh; OK; Good; Great; Terrific] [dropped]
  - My parents
  - My closest friends
  - My siblings
  - School friends/acquaintances

Purpose items:

1. In the past few minutes, to what extent are your actions aligned with your values? [dropped]
2. To what extent do you view what you’re doing *right now* as personally fulfilling?
  - Revised to: To what extent do you view what you’re doing in the past 10 min as personally fulfilling?
3. How much fulfillment do you feel *in this moment*?
  - Revised to: How much fulfillment did you feel in the past 10 min?
4. Do you see your personal values reflected in how you are engaging in the activity you’ve been doing these past few minutes?
  - Revised to: Do you see your personal values reflected in the activity you’ve been doing these past 10 min?
5. How do you view what you’ve been doing in the past few minutes? [response scale from meaningless to meaningful]
  - Revised to: How do you view what you’ve been doing in the past 10 min? [Response options: Extremely meaningless, Somewhat meaningless, Neutral, Somewhat meaningful, Extremely meaningful]

### ESM instructions

All the questions in this mini-survey will ask you about the past 10 min—please answer them considering the past 10 min BEFORE you began this survey.

### ESM set 1

1. What is the strongest emotion you have been feeling in the past 10 min?*

Response options: Happy, Sad, Stressed, Bored, Angry, Scared, Upset, Disgusted, Calm, Excited, Other (Please describe).

1. Item 2: How much did you notice how you were feeling emotionally before we asked?

Response options: Not at all aware, A little aware, Somewhat aware, Very aware, Extremely aware.

Items 3–5 (random order):

1. How connected to others did you feel in the past 10 min?

Response options: Not at all connected, A little connected, Somewhat connected, Very connected, extremely connected.

1. In the past 10 min, did you understand why you think and feel the way you do?

Response options: Not at all aware, A little aware, Somewhat aware, Very aware, Extremely aware.

1. In the past 10 min, how much did you feel nervous or stressed?

Response options: Extremely stressed, Very stressed, Somewhat stressed, A little stressed, Not at all stressed.

#### ESM set 2

(A3) Item 1: How distracted were you in the past 10 min?

Response options: Extremely distracted, Very distracted, Somewhat distracted, A little distracted, Not at all distracted

(A4) Item 2: Had you noticed you were feeling this way before we asked?

Response options: Not at all aware, A little aware, Somewhat aware, Very aware, Extremely aware.

Items 3–5 (random order):

(C2) In the past 10 min, how often did you feel you were supported by others?

Response options: Not at all supported, Occasionally supported, Somewhat often supported, Very often supported, Extremely often supported.

1. To what extent do you view what you’re doing in the past 10 min as personally fulfilling?

Response options: Not at all fulfilling, A little fulfilling, Somewhat fulfilling, Very fulfilling, Extremely fulfilling.

1. In the past 10 min, how much did you feel nervous or stressed?

Response options: Extremely stressed, Very stressed, Somewhat stressed, A little stressed, Not at all stressed.

#### ESM set 3

1. How intense was the emotion that you were feeling in the past 10 min?*

Response options: Not at all intense, A little intense, Somewhat intense, Very intense, Extremely intense.

(A2) Item 2: How aware were you of how you were feeling before we asked?

Response options: Not at all aware, A little aware, Somewhat aware, Very aware, Extremely aware.

Items 3–5 (random order):

(I2) In the past 10 min, did you recognize how your feelings were influencing your outlook on things?

Response options: Not at all aware, A little aware, Somewhat aware, Very aware, Extremely aware.

(P2) How do you view what you’ve been doing in the past 10 min?

Response options: Extremely meaningless, Somewhat meaningless, Neutral, Somewhat meaningful, Extremely meaningful.

1. In the past 10 min, how much did you feel nervous or stressed?

Response options: Extremely stressed, Very stressed, Somewhat stressed, A little stressed, Not at all stressed.

#### ESM set 4

Items 1–5 (all randomized).

(C3) In the past 10 min, how often did you feel connected to others?

Response options: Not at all connected, Occasionally connected, Somewhat often connected, Very often connected, Extremely often connected.

(I3) Did you recognize how your thoughts and feelings were influencing your behavior before we asked?

Response options: Not at all aware, A little aware, Somewhat aware, Very aware, Extremely aware.

(P3) How much fulfillment did you feel in the past 10 min?

Response options: Not at all fulfilled, A little fulfilled, Somewhat fulfilled, Very fulfilled, Extremely fulfilled.

(P4) Do you see your personal values reflected in the activity you’ve been doing these past 10 min?

Response options: Not at all reflected, Occasionally reflected, Somewhat often reflected, Very often reflected, Extremely often reflected.

1. In the past 10 min, how much did you feel nervous or stressed?

Response options: Extremely stressed, Very stressed, Somewhat stressed, A little stressed, Not at all stressed.

*Note: These items are context questions and not used directly as wellbeing measures in the current study.

## References

[1] Kessler, R. C., Chiu, W. T., Demler, O. & Walters, E. E. Prevalence, severity, and comorbidity of twelve-month DSM-IV disorders in the National Comorbidity Survey Replication (NCS-R). *Arch. Gen. Psychiatry* **62**, 617–627 (2005). 15939839 10.1001/archpsyc.62.6.617 PMC2847357

[2] Christie, K. A. et al. Epidemiologic evidence for early onset of mental disorders and higher risk of drug abuse in young adults. *Am. J. Psychiatry* **145**, 971–975 (1988). 3394882 10.1176/ajp.145.8.971

[3] Wittchen, H. U., Nelson, C. B. & Lachner, G. Prevalence of mental disorders and psychosocial impairments in adolescents and young adults. *Psychol. Med.* **28**, 109–126 (1998). 9483687 10.1017/s0033291797005928

[4] Hirshberg, M. J., Heyroth, M. & Davidson, R. J. Wellbeing skills strengthening as a model for healthy adolescent digital technology use. *Ann. N. Y. Acad. Sci.* **1550**, 5–13 (2025). 40729672 10.1111/nyas.15414 PMC12313099

[5] Dahl, R. E. Adolescent brain development: A period of vulnerabilities and opportunities. Keynote address.. *Ann. N. Y. Acad. Sci.* **1021**, 1–22 (2004). 15251869 10.1196/annals.1308.001

[6] Casey, B., Jones, R. M. & Hare, T. A. The adolescent brain.. *Ann. N. Y. Acad. Sci.* **1124**, 111–126 (2008). 18400927 10.1196/annals.1440.010 PMC2475802

[7] Huang, S., Lai, X., Li, Y., Cui, Y. & Wang, Y. Beyond screen time: The different longitudinal relations between adolescents’ smartphone use content and their mental health. *Children* **10**, 770 (2023). 37238318 10.3390/children10050770 PMC10217010

[8] Santos, R. M. S. et al. The associations between screen time and mental health in adolescents: a systematic review. *BMC Psychol.* **11**, 127 (2023). 37081557 10.1186/s40359-023-01166-7 PMC10117262

[9] Orben, A., Meier, A., Dalgleish, T. & Blakemore, S.-J. Mechanisms linking social media use to adolescent mental health vulnerability. *Nat. Rev. Psychol.* **3**, 407–423 (2024).

[10] The teen mental health crisis: a reckoning for Big Tech. https://www.ft.com/content/77d06d3e-2b9f-4d46-814f-da2646fea60c.

[11] Kids’ mental health is in crisis. Here’s what psychologists are doing to help. https://www.apa.orghttps://www.apa.org/monitor/2023/01/trends-improving-youth-mental-health.

[12] van Dam, L., van Os, J., Stams, G. J. & Ormel, H. Alarm bells or echoes of hope? A new perspective on the global youth mental health crisis. *Psychol. Med.* **55**, e332 (2025). 41177561 10.1017/S0033291725102249 PMC13058640

[13] Anderson, M., Faverio, M. & Park, E. *How Teens and Parents Approach Screen Time*. https://www.jstor.org/stable/resrep58219 (2024).

[14] Nagata, J. M. et al. Screen time use among us adolescents during the COVID-19 pandemic: Findings from the Adolescent Brain Cognitive Development (ABCD) Study. *JAMA Pediatr.* **176**, 94–96 (2022). 34724543 10.1001/jamapediatrics.2021.4334 PMC8561427

[15] Trull, T. J. & Ebner-Priemer, U. W. Using experience sampling methods/ecological momentary assessment (ESM/EMA) in clinical assessment and clinical research: Introduction to the special section. *Psychol. Assess.* **21**, 457–462 (2009). 19947780 10.1037/a0017653 PMC4255457

[16] Brose, A., Schmiedek, F., Koval, P. & Kuppens, P. Emotional inertia contributes to depressive symptoms beyond perseverative thinking. *Cogn. Emot.* **29**, 527–538 (2015). 24820350 10.1080/02699931.2014.916252

[17] Zietse, J. et al. Daily resilience: A systematic review of measures and associations with well-being and mental health in experience sampling studies. *Dev. Psychopathol.* **38**, 130–155 (2026). 10.1017/S0954579425000197 40259775

[18] Weber, J., Angerer, P. & Apolinário-Hagen, J. Physiological reactions to acute stressors and subjective stress during daily life: A systematic review on ecological momentary assessment (EMA) studies. *PLoS ONE* **17**, e0271996 (2022). 35895674 10.1371/journal.pone.0271996 PMC9328558

[19] Mölsä, M. E., Lax, M., Korhonen, J., Gumpel, T. P. & Söderberg, P. The experience sampling method in monitoring social interactions among children and adolescents in school: A systematic literature review. *Front. Psychol.* **13** (2022). 10.3389/fpsyg.2022.844698 PMC9013852 35444596

[20] Reitsema, A. M., Jeronimus, B. F., van Dijk, M. & de Jonge, P. Emotion dynamics in children and adolescents: A meta-analytic and descriptive review. *Emotion* **22**, 374–396 (2022). 34843305 10.1037/emo0000970

[21] Ryff, C. D. Eudaimonic well-being. In *Diversity in Harmony—Insights from Psychology* 375–395 (Wiley, 2018). 10.1002/9781119362081.ch20.

[22] Ryan, R. M. & Deci, E. L. On happiness and human potentials: A review of research on hedonic and eudaimonic well-being. *Annu. Rev. Psychol.* **52**, 141–166 (2001). 11148302 10.1146/annurev.psych.52.1.141

[23] Disabato, D. J., Goodman, F. R., Kashdan, T. B., Short, J. L. & Jarden, A. Different types of well-being? A cross-cultural examination of hedonic and eudaimonic well-being. *Psychol. Assess.* **28**, 471–482 (2016). 26348031 10.1037/pas0000209

[24] Kral, T. R. A. et al. Healthy Minds Index: A brief measure of the core dimensions of well-being. *PLoS ONE* **19**, e0299352 (2024). 38728238 10.1371/journal.pone.0299352 PMC11086875

[25] Dahl, C. J., Wilson-Mendenhall, C. D. & Davidson, R. J. The plasticity of well-being: A training-based framework for the cultivation of human flourishing.. *Proc. Natl. Acad. Sci. U. S. A.*10.1073/pnas.2014859117 (2020). 33288719 10.1073/pnas.2014859117 PMC7768706

[26] Hirshberg, M. J., Dahl, C. J., Bolt, D., Davidson, R. J. & Goldberg, S. B. Psychological mediators of reduced distress: Preregistered analyses from a randomized controlled trial of a smartphone-based well-being training.. *Clin. Psychol. Sci.*10.1177/21677026241233262 (2024). 40041238 10.1177/21677026241233262 PMC11877121

[27] Goldberg, S. B. et al. Testing the efficacy of a multicomponent, self-guided, smartphone-based meditation app: Three-armed randomized controlled trial (2020). 10.2196/23825 PMC7732708 33245288

[28] Ryff, C. D. & Keyes, C. L. M. The structure of psychological well-being revisited. *J. Pers. Soc. Psychol.* **69**, 719–727 (1995). 7473027 10.1037//0022-3514.69.4.719

[29] Madl, T. Exploring neural markers of dereification in meditation based on EEG and personalized models of electrophysiological brain states. *Sci. Rep.* **14**, 24264 (2024). 39414816 10.1038/s41598-024-73789-8 PMC11484965

[30] Mey, L. K. et al. Be kind to yourself: The implications of momentary self-compassion for affective dynamics and well-being in daily life. *Mindfulness* **14**, 622–636 (2023). 36644400 10.1007/s12671-022-02050-y PMC9823261

[31] Hanley, A., Dorjee, D. & Garland, E. Mindfulness training encourages self-transcendent states via decentering. *Psychol. Conscious. Theory Res. Pract.* **10**, 431–440 (2020).

[32] Song, J., Howe, E., Oltmanns, J. R. & Fisher, A. J. Examining the concurrent and predictive validity of single items in ecological momentary assessments. *Assessment* **30**, 1662–1671 (2023). 36004406 10.1177/10731911221113563 PMC10248304

[33] Chajut, E. & Algom, D. Selective attention improves under stress: Implications for theories of social cognition. *J. Pers. Soc. Psychol.* **85**, 231–248 (2003). 12916567 10.1037/0022-3514.85.2.231

[34] van Marle, H. J. F., Hermans, E. J., Qin, S. & Fernández, G. From specificity to sensitivity: How acute stress affects amygdala processing of biologically salient stimuli. *Biol. Psychiatry* **66**, 649–655 (2009). 19596123 10.1016/j.biopsych.2009.05.014

[35] Maydych, V., Claus, M., Watzl, C. & Kleinsorge, T. Attention to emotional information is associated with cytokine responses to psychological stress. *Front. Neurosci.* **12**, (2018). 10.3389/fnins.2018.00687 PMC6176093 30333720

[36] Cohen, S., Kamarck, T. & Mermelstein, R. A global measure of perceived stress. *J. Health Soc. Behav.* **24**, 385–396 (1983). 6668417

[37] Cheung, F. & Lucas, R. E. Assessing the validity of single-item life satisfaction measures: Results from three large samples. *Qual. Life Res.* **23**, 2809–2818 (2014). 24890827 10.1007/s11136-014-0726-4 PMC4221492

[38] Topp, C. W., Østergaard, S. D., Søndergaard, S. & Bech, P. The WHO-5 Well-Being Index: A systematic review of the literature. *Psychother. Psychosom.* **84**, 167–176 (2015). 25831962 10.1159/000376585

[39] Watson, D., Clark, L. & Tellegen, A. Development and validation of brief measures of positive and negative affect: The PANAS scales. *J. Pers. Soc. Psychol.* **54**, 1063–1070 (1988). 3397865 10.1037//0022-3514.54.6.1063

[40] Spielberger, C. D., Auerbach, S. M., Wadsworth, A. P., Dunn, T. M. & Taulbee, E. S. Emotional reactions to surgery. *J. Consult. Clin. Psychol.* **40**, 33–38 (1973). 4688678 10.1037/h0033982

[41] Johnson, C., Burke, C., Brinkman, S. & Wade, T. Development and validation of a multifactor mindfulness scale in youth: The Comprehensive Inventory of Mindfulness Experiences-Adolescents (CHIME-A). *Psychol. Assess.* **29**, 264–281 (2017). 27254018 10.1037/pas0000342

[42] Kesebir, P., Gasiorowska, A., Goldman, R., Hirshberg, M. J. & Davidson, R. J. Emotional style questionnaire: A multidimensional measure of healthy emotionality. *Psychol. Assess.* **31**, 1234–1246 (2019). 31259572 10.1037/pas0000745 PMC6776667

[43] Shiota, M. N., Keltner, D. & John, O. P. Positive emotion dispositions differentially associated with Big Five personality and attachment style. *J. Posit. Psychol.* **1**, 61–71 (2006).

[44] Gross, J. J. & John, O. P. Individual differences in two emotion regulation processes: Implications for affect, relationships, and well-being. *J. Pers. Soc. Psychol.* **85**, 348–362 (2003). 12916575 10.1037/0022-3514.85.2.348

[45] Francis, L. J. Implicit religion, explicit religion and purpose in life: An empirical enquiry among 13- to 15-year-old adolescents. *Ment. Health. Relig. Cult.* **16**, 909–921 (2013).

[46] Costin, V. & Vignoles, V. L. Meaning is about mattering: Evaluating coherence, purpose, and existential mattering as precursors of meaning in life judgments. *J. Pers. Soc. Psychol.* **118**, 864–884 (2020). 30614732 10.1037/pspp0000225

[47] Hox, J., Moerbeek, M. & Van de Schoot, R. *Multilevel Analysis: Techniques and Applications* 3rd edn. (Routledge, 2017). 10.4324/9781315650982.

[48] Nakagawa, S., Johnson, P. C. D. & Schielzeth, H. The coefficient of determination R2 and intra-class correlation coefficient from generalized linear mixed-effects models revisited and expanded. *J. R. Soc. Interface* **14**, 20170213 (2017). 28904005 10.1098/rsif.2017.0213 PMC5636267

